# Is cough really necessary for TB transmission?

**DOI:** 10.1016/j.tube.2019.05.003

**Published:** 2019-07

**Authors:** Benjamin Patterson, Robin Wood

**Affiliations:** a University of Amsterdam, Amsterdam Institute for Global Health and Development, Amsterdam, the Netherlands; b Desmond Tutu HIV Centre, IDM, University of Cape Town, Cape Town, South Africa; c Institute of Infectious Disease and Molecular Medicine (IDM), Faculty of Health Sciences, University of Cape Town, Cape Town, South Africa

## Abstract

Cough has long been implicated in the production of infectious aerosol leading to transmission of tuberculosis (TB). However, prevalence studies frequently identify radiographic evidence of TB in subclinical individuals in the absence of reported coughing. Elucidating the role of cough in transmission depends on understanding the physical process of aerosolizing and expelling mycobacterium tuberculosis (Mtb) bacilli. In the last decade, human aerosol studies have progressed with improved precision of particle detection and greater sophistication of experimental protocols. Combining principles of respiratory physiology, the site and mechanism of aerosolization of respiratory lining fluids during phases of the respiratory cycle has been investigated in detail. Additionally, recent success in the direct detection of naturally generated Mtb aerosols has allowed more detailed characterization in terms of their rate of production and size distribution. We propose that TB transmission depends on the coincidence of the site of aerosol generation with the presence of Mtb bacilli. This review will examine the evidence for site of aerosol production during cough and respiratory activities in conjunction with the characteristics of detectable Mtb aerosols and locations of tuberculosis infection. Furthermore, we propose respiratory activities that are likely to optimise aerosol sampling for investigation of transmission.

## 1 Introduction

Cough is often assumed to be a primary mechanism for transmission of *Mycobacterium tuberculosis* [1]. A frequent and prominent symptom of pulmonary TB, cough, now of any duration in high risk individuals should trigger screening according to the updated American College of Chest Physicians (ACCP) clinical practice guideline [2]. Persistent cough evoked by stimulation of bronchopulmonary C-fibres and airway mechanoreceptors may indicate bronchopulmonary disease [3]. Evidence exists that cough frequency correlates with individual TB infectivity [[4], [5], [6]]. Indeed, the centrality of cough in TB transmission has long-standing support in the research literature [7] leading to the promotion of cough etiquette [8] and advocacy for antitussive agents [9] as transmission control measures. However, direct evidence for cough as the physical mechanism for *Mtb* bacillus expulsion and onward transmission is lacking.

Knowledge of human respiratory aerosol production has improved markedly in recent years with an evolving consensus on the sites in the respiratory tract where aerosol is generated. All breathing activity produces aerosol but the aerosol cloud generated from quiet tidal breathing can be greatly increased with certain respiratory manoeuvres [[18], [19], [20], [21]]. Most recent, sophisticated studies have investigated aerosol generated from healthy subjects [[17], [18], [19], [20], [21], [22], [23]] but it is likely that the principles identified remain valid in disease states since the anatomical structures are unchanged.

TB is known to be transmitted by airborne infectious aerosol in the 1–5 μm range which are small enough to remain airborne and disperse on air currents. Numerous aerosol-generating respiratory activities have been implicated in transmission including coughing [10,11], sneezing [11], singing [12,13] and talking [11,13]. However, evidence for the relative importance of these different respiratory activities is largely circumstantial based on outbreak investigations and observed associations.

Parallel developments in air sampling systems have achieved direct detection of viable *Mtb* aerosols after 10-min of voluntary coughing into a cough box [14], 1-h of natural respiratory activity [5] inside a sealed chamber, and 10–300-min of natural respiratory activity while wearing a sampling mask by individuals with pulmonary TB [15]. The size distribution for infectious *Mtb* aerosol has been demonstrated by use of a 6-stage Andersen impactor with good agreement between two studies [5,14].

This review will examine the evidence for aerosol production in the small peripheral airways, large airways and laryngeal structures and discuss the relative importance during different respiratory activities. We also evaluate the evidence for production of aerosol in TB disease and the characteristics of detectable *Mtb* aerosols. Additionally, we review the indirect evidence for the role of cough in generating infectious aerosol.

## 2 Methods

In this narrative review, we have restricted our focus to themes of human aerosol production, direct studies characterising infectious TB aerosols and association between cough and TB. We did not include papers that were not relevant to these themes. We conducted several separate searches for original articles and reviews published in English up to 31st December 2018 with combinations of keywords including: human expired aerosol, size distribution, mechanism, cough, mycobacterium, tuberculosis. Medline, Embase and Scopus databases were used to search the literature. Articles from our personal collections were also included.

### 2.1 Aerosol-generating activities

Multiple respiratory activities produce aerosol both in health and disease from several distinct locations between the alveoli and the mouth. Identifying the events that lead to production of an infectious particle responsible for a specific infection is near impossible. However, aspects of the production of viable (culturable) bacilli in the respirable range (1–5 μm) can be elucidated and the potential for onward transmission may reasonably be inferred. It seems highly plausible that aerosol derived from sites adjacent to infected tissue have the greatest likelihood to bear pathogen [16]. Identifying the locations of aerosol production is therefore central to determining relative importance of respiratory activities in transmission. Size of aerosol produced is also significant since larger aerosols (>5 μm) settle rapidly in the environment and are therefore of negligible importance for organism transmission. Our focus, in this review, will be on small aerosols (<5 μm) and will not include discussion of aerosol derived from the mouth.

### 2.2 Sites of aerosol production

Numerous studies conducted on healthy individuals have identified several aerosol sources within the respiratory tract [[16], [17], [18], [19], [20], [21], [22], [23]]. Both site and mechanism of generation have also been elucidated through careful standardisation of respiratory manoeuvres. We discuss three described sites of aerosol generation.

#### 2.2.1 Bronchiole aerosol

**Fig. 1:**
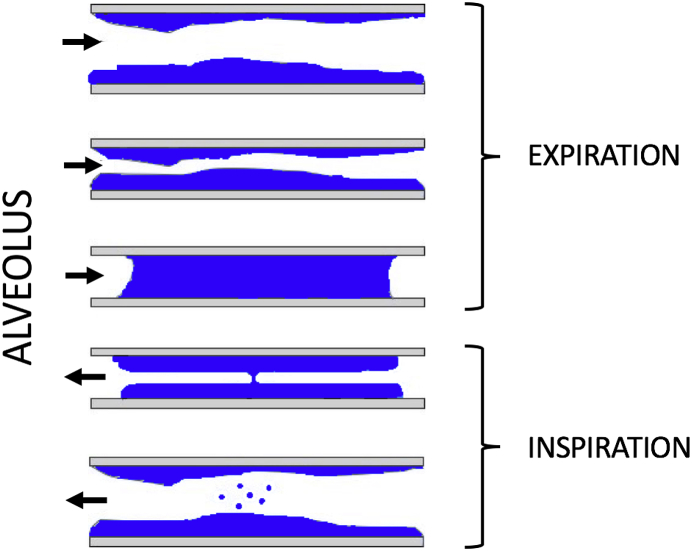
Aerosol creation in terminal bronchioles by the bronchiole fluid film burst mechanism (Modified from Johnson et al. ref 18).

The generation of aerosol during tidal breathing has been debated. Over recent years, consensus has emerged around a mechanism termed bronchiole fluid film burst (BFFB) (see Fig. 1) [18,[20], [21], [22],^24^]. This occurs following small airway ‘fluid’ closure in terminal bronchioles during expiration. In early inspiration, the expanding bronchiole stretches the fluid blockage creating a film which bursts to form aerosol. This aerosol is drawn into alveoli in late inspiration prior to expiration.

Reproducible features of respiratory aerosol production during tidal breathing were first identified by Gebhart et al. [25] namely: (1) expired particle concentration increases with tidal volume when inspiration starts at airway closing volumes; (2) respiratory flow rate is of minor influence on expired particle concentration; (3) breath-holding decreases the expired particle content; (4) simultaneous measurement of CO_2_ concentration shows that expired particles are from volumetric lung depths >200 cm^3^ (volumetric lung depth refers to the volume of air exhaled between the start of exhalation and detection of expired particles).^1^ (5) subjects with collapsing airways have an increased production rate of particles.

**Fig. 2:**
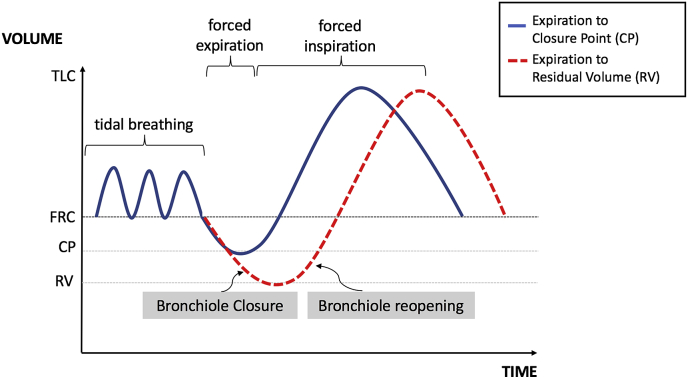
Schematic representation of the respiratory cycle illustrating the closure point (CP) between the functional residual capacity (FRC) and the residual volume (RV). The CP volume can be experimentally identified using the ^133^Xe bolus method [26] and indicates the depth of expiration when airways closure begins. Terminal bronchiole closure and subsequent reopening occurs most prominently at expiratory volumes beyond the closure point. The blue line indicates expiration to CP and the red line indicates expiration beyond CP leading to greater aerosol production (Modified from Almstrand A et al. ref. 20). (For interpretation of the references to colour in this figure legend, the reader is referred to the Web version of this article.)

Re-opening of small airways explains these five key features and is supported by recent investigations. Johnson et al. conducted a series of experiments altering breathing patterns in terms of length and depth of inspiration and expiration in healthy subjects [18]. A deep expiration preceding a breath (increasing the number of fluid blocked bronchioles) raised expired particle concentration by a factor of 5.5. Similar studies have replicated this finding [20,21]. In particular, higher aerosol counts were recorded when expiration occurred at greater depth than the terminal bronchiole *closure point* (see Fig. 2.) [20]. Rapid expiration produced no more particles compared with slow expiration suggesting respiratory fluid shearing is not occurring during tidal breathing. Reduction in expired aerosol content with breath-holding is explained by aerosol formation during *inspiration* and subsequent gravitational settling in the alveoli. This is also consistent with time dependence of the reduction in aerosol and preferential loss of the larger particles [18]. Finally, a consistent correlation between age and aerosol production is likely due to the loss of elastic recoil causing greater propensity for airway closure in older individuals [18,21].

#### 2.2.2 Bronchial aerosol

**Fig. 3:**
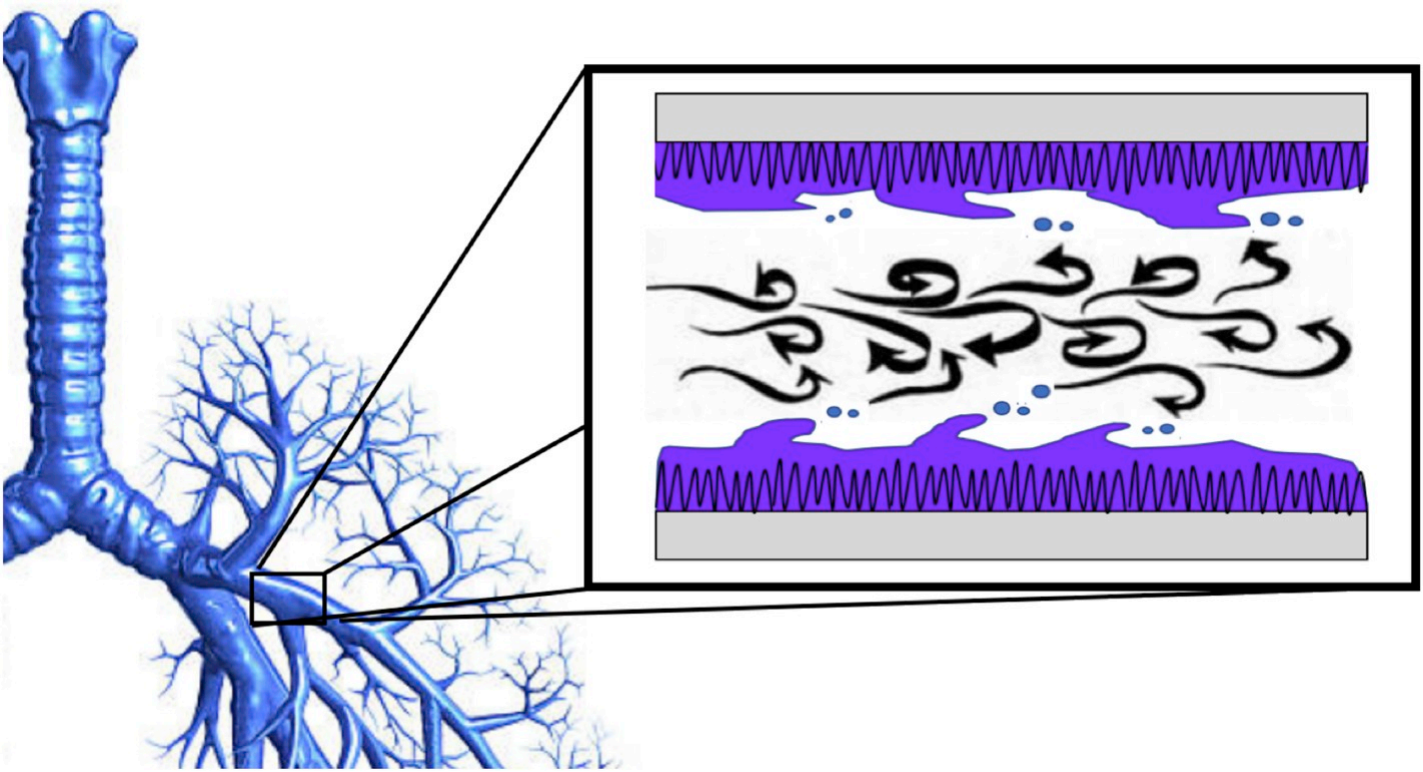
Illustration of aerosol creation by shear forces in large bronchi.

Aerosol may also be produced by shear forces destabilising the mucous/air surface (see Fig. 3). The highest velocity air flow in the respiratory tract is at the point of lowest total cross-sectional area which corresponds to the 8th generation of airway branching [27]. Airflow is most prone to become turbulent at this point and high expiratory flow rates (up to ∼50 msec^−1^) during coughing and sneezing are most likely to cause turbulence. Experimental simulations of cough through rigid tubes with simulated mucous have been developed largely to model clearance of mucous and have not investigated aerosol production [[28], [29], [30]]. One study, however, used a rectangular acrylic model trachea lined on the bottom surface with a mucous simulant to evaluate the effect of changing surface tension properties with inspired saline and surfactant. Passing a rapid air puff through the tube mimicked a cough and led to production of only minimal aerosol of 0.3 μm median size. Only following nebulization of isotonic saline into the tube did the median aerosol size increase to the respirable range (1–5 μm) [31].

The fluid mechanical considerations for aerosol production from a branching system of compressible tubes lined with non-Newtonian fluid are highly complex [32]. Multiple factors affect aerosol generation including cough velocity, mucous density, surface tension and viscosity. The lack of direct empirical evidence confirming aerosol production from this mechanism is problematic for assuming its prominence in TB transmission.

#### 2.2.3 Laryngeal aerosol

**Fig. 4:**
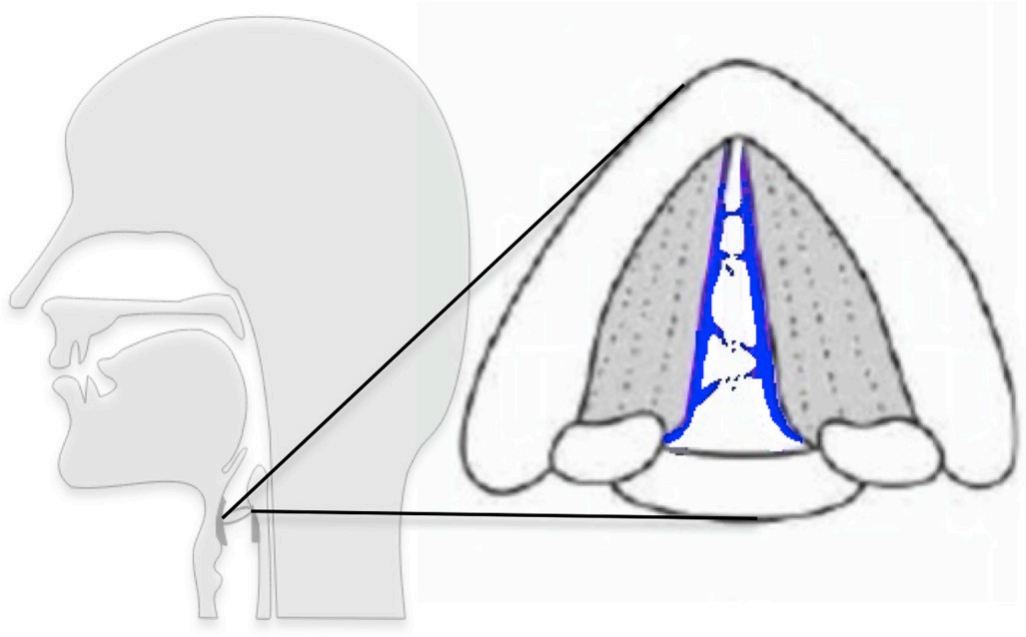
Laryngeal aerosol creation by vocal fold vibration and closure and reopening cycles.

Laryngeal structures have also been found to generate aerosol. This component of breath aerosol is common to noise-producing respiratory manoeuvres. Aerosol in a consistent size distribution can be accentuated by selectively creating energetic vocal fold vibrations with sustained vocalisation (prolonged “aah”) [19]. This same ‘laryngeal mode’ is also produced by coughing suggesting a correspondence to aerosol generated by vocal fold adduction. Potentially close apposition of the vocal folds produces liquid bridges of mucous that burst creating aerosol (see Fig. 4). TB infection of the larynx was frequently diagnosed in the pre- and early chemotherapeutic era where the combination of local pathogen and laryngeal aerosol production resulted in extremely high transmission risk.

Of interest Johnson et al. [19] explain aerosol production from cough without recourse to a bronchial source (B). By fitted a finite mixture model to expired aerosol size distributions from breathing, speaking, vocalisation and coughing they identify several overlapping modes [17,19]. These modes are present to varying degrees with different respiratory activities and comprise a bronchiole mode (source A), a laryngeal mode (source C) and an additional oral mode (mouth, lips and tongue).

### 2.3 Aerosol sources in different respiratory activities

Aerosol derived from respiratory lining fluid during different respiratory activities comprises a combination of these sources (see Table 1). Tidal breathing typically produces a small concentration of aerosol from the bronchiole source (A), this is likely also the case for yawning although with deeper inspiration and expiration. Coughing, sneezing and vocalising (speech, singing) produce larger concentrations of aerosol derived from both bronchiole and laryngeal sources (A & C). High expiratory flow rate manoeuvres such as coughing and sneezing may additionally lead to aerosolization due to shear stress from larger airways (B). However, empirical evidence to support this mechanism is less well established.

**Table 1:** Indicates probable mechanisms involved in generating small (1–5 μm) aerosol during the various respiratory manoeuvres. Aerosol from BFFB arising in the terminal bronchioles (A), bronchial aerosol arising from the large airways (B) and laryngeal aerosol arising from the larynx and vocal folds (C).

	Respiratory Activity	Aerosol Mechanism			Exhaled Volume (L) (approx.)	Frequency (min^−1^) (approx.)
		A	B	C		
*NATURAL PRODUCTION*	Tidal breath	+	–	–	0.5	12
	Cough	+	+/−	+	1–1.5	0.5^a^
	Sneeze	+	+/−	+	1.5	infrequent
	Speech	+	–	+	0.5	intermittent
	Singing	+	–	+	1	intermittent
	Yawn	+	–	–	2	infrequent
*DIAGNOSTIC*	Cough	+	+/−	+	1.5	3 (uncomfortable)
	FEV_1_	+	+/−	–	4	3 (uncomfortable)
	Slow FVC	+	–	–	5	6–10

a [Ref 5].

Aerosol sampling as a diagnostic tool depends on identifying voluntary respiratory activities which produce aerosol from the relevant region of the respiratory tract. Pathogens of the upper airway are likely to be contaminate aerosol generated from the laryngeal source (C). Lower airway disease, on the other hand, is more likely to contaminate bronchiole derived aerosol (A).

### 2.4 Aerosol production in TB disease

The quantity, site of production and size distribution of aerosol in TB disease has not been extensively studied. Particle counts in individuals with pulmonary TB disease are known to vary widely but no correlation has been found with increased production and presence of culturable *Mtb* in aerosol [5]. Increased odds of high aerosol production in the 1–5 μm range during tidal breathing has been found in patients with intrathoracic TB compared with extra-pulmonary TB and healthy controls [33]. The same study also found an association between aerosol and increased age in keeping with the BFFB mechanism for aerosol production in this size range. Conceivably infected lung tissue may have a greater propensity for airway closure leading to greater aerosol creation by BFFB.

### 2.5 Aerosolized *Mtb*

Two studies used an Andersen impactor to demonstrate viable (culturable) bacilli in aerosol ranging in size from 0.5 μm to 8.2 μm [4,5]. Both studies identified greater numbers of organisms at impactor stages 4–5 (corresponding to aerosol diameters 1.0–3.5 μm). Of note, this was found to approximately match the relative size distribution of simultaneously measured aerosol [5]. The fact that the lower bound of the size range is smaller than the estimated length of the bacillus is perhaps explained by the bacillus having a smaller aerodynamic diameter.

### 2.6 Cough frequency in TB disease

Quantitative cough assessment and TB disease has been rarely studied. In 1969 Loudon and Spohn recorded 8-h nocturnal cough counts (pre-treatment mean of 13.6 coughs/hour) showing that the frequency of coughing steadily decayed over successive weeks on anti-tuberculosis chemotherapy [13]. More recently a study in Peru found a substantially lower pre-treatment median of 2.3 coughs/hour but replicated the treatment effect on cough frequency with reductions predicting microbiological conversion of sputum [34]. The same group showed a link between greater cough counts as well as persistent coughing on treatment and the presence of larger cavity volumes and proximity of cavities to airways [35]. These findings indicate a correlation between coughing and disease extent but cannot be taken as evidence for cough as a means of transmission. Another recent study in the UK measured 24-h cough frequencies and found that cough frequency of the index cases was associated with infection in household contacts [6].

Further insight for the role of cough in TB transmission comes from aerosol sampling studies. Analysis of sound recordings over 1-h of patient sampling in the Respiratory Aerosol Sampling Chamber (RASC) found a higher median cough count in patients with a positive aerosol culture (12 coughs/hour compared with 26 coughs/hour) suggesting that frequently coughing patients may be more infectious [5]. However, conflicting with this, one patient sampled in the RASC was noted to have detectable *Mtb* genome equivalents by ddPCR in the absence of any recorded coughs. This significant finding has also been replicated in a mask aerosol sampling study. Intermittent wearing of an FPP1 mask modified with a gelatine filter detected *Mtb* aerosol by PCR in 22 out of 24 newly diagnosed TB patients over a 24-h period. 11 samples from these patients were positive for *Mtb* in the absence of recordable coughs. [36 - preprint].

### 2.7 Role of respiratory activities in TB transmission

Aerosol production from the laryngeal source (C) is unlikely to play a prominent role in transmission since pulmonary TB disease is remote from laryngeal structures. Laryngeal TB (1–2% of cases [37]) is an exception and the well-recognized high infectivity [38] may be explained by large volumes of laryngeal aerosol efficiently generated by speech and coughing.

Most cases of TB disease are likely to be spread by either aerosol from bronchiole (A) or bronchial (B) sources. Bronchial aerosol, if present, would occur during high flow expiratory events like coughing and sneezing. In contrast, bronchiole aerosol is common to all breathing activity but becomes significantly more prominent when the depth of expiration exceeds the airway closure point. Therefore, infected aerosol is likely to be emitted during singing, talking or coughing and to a lesser extent during tidal breathing. A single cough therefore has an increased likelihood to carry *Mtb* aerosols compared with a single tidal breath due to depth of the preceding expiration and increased bronchiole aerosol content. However, since breathing is typically at least 30 times more frequent than coughing in TB disease (median 26 cough/hour [5], approx. 720 breathes/hour) *cough Mtb* aerosols may be rarer than *breath Mtb* aerosols.

## 3 Conclusion

Coughing does not appear to be a necessary prerequisite for TB transmission. All respiratory activities potentially release aerosol from sites of TB disease in the lung via the bronchiole fluid film burst mechanism. This mechanism is present, to varying degrees, with every breath and has characteristics which suggest a prominent role in generating *Mtb*-laden aerosol. Evidence from human aerosol studies indicate that this mechanism alone leads to aerosol release from the lung periphery where TB disease is commonly located. These small airways may also communicate with cavities in the lung parenchyma where bacilli are concentrated [39]. Further evidence comes from the consistently high sensitivity of broncho-alveolar lavage in TB, even in cases with negative sputum culture [40,41]. This suggests that viable *Mtb* bacilli are to be found in the lower airways.

Frequency of cough is associated with infectivity but this does not imply mechanistic causality for TB transmission. A greater burden of airway disease can lead to both the production of higher concentrations of *Mtb* aerosol as well as frequent coughing. This is further supported by the important finding that aerosolized TB has been detected by PCR in exhaled air from patients who have not coughed at all during a sampling period [5,36]. Importantly, the absence of cough should therefore not be reassuring for lack of infectivity. Moreover, a research goal should be to more fully understand which respiratory manoeuvres generate *Mtb* bacillus-laden aerosol and from which anatomical locations. Such insight could lead to novel interventions with the potential to interrupt the spread of infection.

The prospect of transmission in the absence of cough may be highly significant. Molecular epidemiology studies indicate that most new and recurrent TB disease in high prevalence settings are the result of recent transmission [42]. The frequent observation in the same settings that only 1–30% of new *Mtb* infections can be linked with known TB cases implies the existence of many unrecognized transmitters in TB endemic communities [43,44]. Non-coughing, sub-clinical individuals as commonly encountered in national TB prevalence surveys (ranging from 40 to 79% of microbiologically confirmed cases [45]) may account for some of these missing transmitters.

## 4 Future directions for aerosol sampling

Aerosol sampling as a tool to identify *Mtb* transmitters has great potential. Development of a functional system requires a focus on three critical areas: (1) the breathing protocol to generate aerosol, (2) maximising aerosol collection and (3) sensitive *Mtb* detection. Research characterising human aerosol production, discussed in this review, is invaluable for determining the appropriate protocol to maximise yield from diseased regions of the respiratory tract. Bronchiole aerosol from peripheral small airways is best generated by increased depth of expiration leading to greater numbers of small airway closures. High expiratory flow rates such as produced by voluntary coughing is poorly tolerated and unlikely to be necessary.

Sufficiently sensitive *Mtb* detection may be best achieved by a non-culture method given the very low numbers of bacilli present. Detection of non-viable or differentially culturable bacilli is therefore highly probable. Further work will be required to establish whether there is equivalent proportion of these organisms in aerosol samples as have been identified in sputum [46].

## Footnotes

1 Volumetric capnography measures breath-by-breath CO_2_ concentration and exhaled air volume. For an individual a reference curve can be created and used to assess lung depth for a given exhaled CO_2_ fraction. A typical expiratory reserve volume (end-tidal volume to reserve volume) is 1000 cm^3^.
